# “A comparison of thermal stress response between *Drosophila melanogaster* and *Drosophila pseudoobscura* reveals differences between species and sexes*”*

**DOI:** 10.1016/j.jinsphys.2024.104616

**Published:** 2024-01-24

**Authors:** N. Rivera-Rincón, U.H. Altindag, R. Amin, R.M. Graze, A.G. Appel, L.S. Stevison

**Affiliations:** Department of Biological Sciences, Auburn University, Auburn, AL USA

**Keywords:** *Drosophila*, Climate change, Fecundity, Thermal tolerance, Oogenesis

## Abstract

The environment is changing faster than anticipated due to climate change, making species more vulnerable to its impacts. The level of vulnerability of species is influenced by factors such as the degree and duration of exposure, as well as the physiological sensitivity of organisms to changes in their environments, which has been shown to vary among species, populations, and individuals. Here, we compared physiological changes in fecundity, critical thermal maximum (CT_max_), respiratory quotient (RQ), and DNA damage in ovaries in response to temperature stress in two species of fruit fly, *Drosophila melanogaster* (25 vs. 29.5 °C) and *Drosophila pseudoobscura* (20.5 vs. 25 °C). The fecundity of *D. melanogaster* was more affected by high temperatures when exposed during egg through adult development, while *D. pseudoobscura* was most significantly affected when exposed to high temperatures exclusively during egg through pupal development. Additionally, *D. melanogaster* males exhibited a decrease of CT_max_ under high temperatures, while females showed an increase of CT_max_ when exposed to high temperatures during egg through adult development. while *D. pseudoobscura* females and males showed an increased CT_max_ only when reared at high temperatures during egg through pupae development. Moreover, both species showed an acceleration in oogenesis and an increase in apoptosis due to heat stress. These changes can likely be attributed to key differences in the geographic range, thermal range, development time, and other different factors between these two systems. Through this comparison of variation in physiology and developmental response to thermal stress, we found important differences between species and sexes that suggest future work needs to account for these factors separately in understanding the effects of constant increased temperatures.

## 1. Introduction

Recent studies on temperature changes have indicated that temperatures are rising at a faster rate than previously predicted. It is expected that temperatures will increase by approximately 1.5 °C between 2023 and 2027 (IPPC, 2007), with particularly concerning temperature spikes, especially during the summer months. Therefore, species must adapt not only to rising mean temperatures but also to pronounced short-term changes in temperature. This is particularly crucial for species with shorter life spans, as they may encounter thermal stressors for more significant portions of their life cycle (Hoffmann et al., 2013; Kingsolver et al., 2013; Parmesan, 2006; Piyaphongkul, 2013). Understanding the effects of thermal stress on species is essential for accurately assessing the impacts of climate change (Chevin et al., 2013; Schilthuizen & Kellermann, 2014; Seebacher et al., 2015).

Increasing temperature is one of the most ubiquitous impacts of climate change and has widespread impacts on life history traits. For example, the response of a species to heat stress is strongly related to growth rates (Feder & Burggren, 1992; Potter et al., 2011), fecundity (Krebs and Loeschcke, 1994), and fertility (Walsh et al., 2019). The degree of vulnerability of an organism to heat stress has been defined as the ability of an organism to maintain fitness and cope with the effects of increasing temperatures (Walsh et al., 2021). For example, the endemic Hawaiian *Drosophila* species has been shown to be more sensitive to small changes in temperature, resulting in significant declines in species diversity, and populations (Uy et al., 2015). Although thermal tolerance has been used to estimate vulnerability to increased temperatures (Huey et al., 2012), the level of vulnerability of species is influenced by other factors such as the degree and duration of exposure, as well as the physiological sensitivity of organisms to changes in their environments (Bernardo et al., 2007; Calosi et al., 2008; Colado et al., 2022; Diamond et al., 2017; Greenspan et al., 2017; Khaliq et al., 2014; Sunday et al., 2012). Thermal tolerance is linked to the magnitude of temperature variation that organisms experience over time (Addo-Bediako et al., 2000; Barley et al., 2021; Ghalambor et al., 2006; Janzen, 1967; Rodgers & Isaza, 2022). Further, thermal tolerance has been observed to be dramatically different among species (Kaspari et al., 2015), populations at different geographic distributions (Nguyen et al., 2019; Rey et al., 2012), and individuals within populations (Logan et al., 2014). Differences in thermal tolerance are present in a wide variety of taxa, including seahorses (Mascaró et al., 2016), snails (Brahim & Marshall, 2020; Gaitan-Espitia et al., 2013; Kuo & Sanford, 2009), fish (Campos et al., 2021; Nyboer & Chapman, 2018; Schaefer & Ryan, 2006), and copepods (Pereira et al., 2017; Sasaki & Dam, 2020). For example, in copepods, populations distributed across a latitudinal thermal gradient varied in survivorship and knockdown temperature under acute thermal exposures. Nevertheless, when the thermal exposure was chronic, survivorship and developmental times were more similar at extreme ranges than in the middle (Harada et al., 2019), suggesting that physiological adaptation occurs at a very local scale.

Insect physiology, development, metabolism, and reproduction are constrained by temperature changes (Colinet et al., 2015; Harrison et al., 2012; Sinclair et al., 2016), making insects suitable bioindicators to monitor the effects of climate change, particularly fruit fly species of the genus *Drosophila* (Parsons, 1989). The effects of thermal stress have been thoroughly quantified (David et al., 2005) in *Drosophila* revealing that they respond adaptively to selection for heat (Hoffmann et al., 1997; Loeschcke & Krebs, 1996; Morrison & Milkman, 1978) and cold stress (Chen & Walker, 1993; Watson & Hoffmann, 1996), exhibiting genetic variability for heat and cold tolerance. The widely studied and cosmopolitan species *Drosophila melanogaster* can be found at temperatures ranging between 11 and 32 °C. While the alpine species *Drosophila pseudoobscura* has a narrower geographic distribution, which is endemic to the Americas, including the Western United States, Mexico, and Bogotá, Colombia (Myers & Frankino, 2012). *D. pseudoobscura* is found in temperatures ranging between 16 and 29 °C, a nearly 40 % lower thermal range than *D. melanogaster*. These two species with different geographic distributions, development times, ecology, and thermal ranges make ideal models to compare physiological and other indicators of thermal stress. The comparison of thermal stress indicators can help to better understand the relationship between thermal stress responses and life history traits among species.

Here, we used various reproductive and physiological assays to measure the response of two different species of *Drosophila* to thermal stress to test whether differences between temperature, species, sex, and life stages, or a combination of these factors are important in these responses. We hypothesized that *D. pseudoobscura*, due to its alpine distribution (Myers & Frankino, 2012), would show a greater response to thermal stress than the cosmopolitan *D. melanogaster* (David & Bocquet, 1975). We further hypothesized that both earlier developmental life stages (Kinzner et al., 2019), and males as compared to females (Van Heerwaarden & Sgro, 2021) would be more susceptible to thermal stress regardless of species. Specifically, we compared changes in fecundity, critical thermal maximum (CT_max_), respiratory quotient (RQ), and DNA damage in ovaries in response to temperature stress in two species of fruit fly, *D. melanogaster* (a cosmopolitan species) and *D. pseudoobscura* (an endemic US species). Considering the differences in life history between both species, species-specific treatment crosses were set up at appropriate temperature ranges in control and high temperatures for *D. melanogaster* (25–29.5 °C) and *D. pseudoobscura* (20.5–25 °C). Additionally, recently eclosed F_1_ flies were either switched to the control temperature for larval stress only or kept at the treatment temperature for larval plus adult stress, to evaluate the effects of temperature on reproduction at different developmental stages, for a total of four different treatment groups. Results from this study will allow for the comparison of physiological and developmental responses to temperature stress between species with different thermal tolerance, geographic distributions, and development time; as well as providing a better understanding of how species may respond to rising temperatures and the potential impacts of climate change.

## 2. Methods

### 2.1. Fly stocks

Temperature stress measurements were conducted using the wildtype inbred stock Canton-S (courtesy of Dr. Michelle Arbeitmen) of *D. melanogaster*, collected from Canton, Ohio, USA, and the wildtype stock MV2–25 (courtesy of Dr. Mohamed Noor) of *D. pseudoobscura*, collected from Mesa Verde, Colorado, USA.

### 2.2. Fly husbandry and cross design

**Fig. 1.**
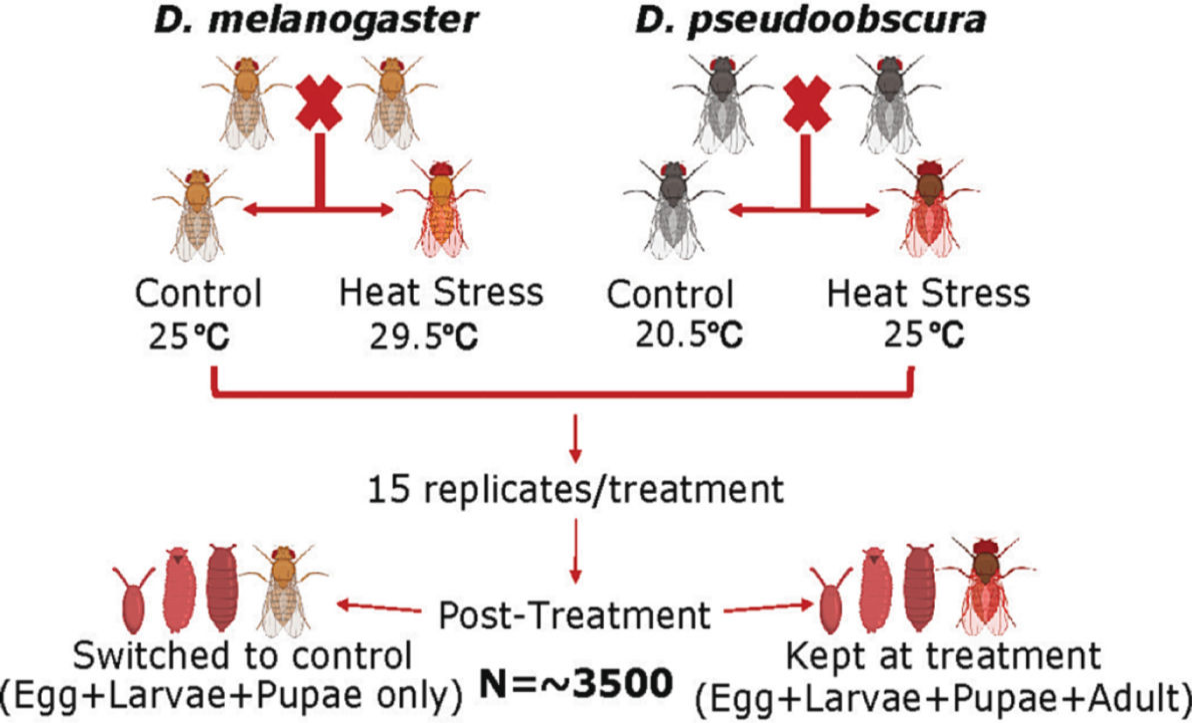
Graphical description of fly husbandry and cross design.

All stocks of *D. melanogaster* and *D. pseudoobscura* were maintained at 25 °C and 20.5 °C, respectively with a photoperiod of 12:12 (light:dark) in incubators. Flies were reared on standard cornmeal-sugar-yeast-agar media in polypropylene enclosures. In each treatment group, 15 replicate crosses were set up with 15 flies approximately, in a 2:1, female-male ratio, aiming for ~ 3500 progeny per species. Parental flies were reared under control conditions and virgin females were collected and held at those conditions until reaching complete sexual maturity, 2 days for *D. melanogaster* and 7 days for *D. pseudoobscura*. Before conducting the experiment, it was noted from previous studies that *D. melanogaster* could tolerate temperatures as high as 30 °C (McKenzie & Parsons, 1974). To determine the highest non-lethal temperature for *D. melanogaster*, preliminary studies were conducted with 0.5 °C temperature increments up to 30 °C. The highest non-lethal temperature for Canton-S *D. melanogaster* where the flies were able to reproduce was 29.5 °C, which was selected for the high temperature treatment. For the control treatment, the optimum temperature of 25 °C for *D. melanogaster* and 20.5 °C for *D. pseudoobscura* was used. The control temperature for *D. melanogaster* was also used as the high temperature for *D. pseudoobscura* to optimize the use of incubator space. F_1_ crosses were reared in high temperature treatment conditions throughout development, 29.5 °C and 25 °C for *D. melanogaster* and *D. pseudoobscura*, respectively (Fig. 1). These treatment temperatures allowed an increase in the temperature by 4.5 °C above the optimal temperature for each species as a standard adjustment. Specifically, we opted to apply a constant temperature shift in each species, as opposed to the same specific temperature, because in nature they occupy different microhabitats based on behavior and environmental preferences (Diepenbrock & Burrack, 2017; Kinzner et al., 2019; Taylor, 1987). While we cannot account for behavioral preference in a laboratory setting, we expect that the shifts in temperature of the micro-habitats due to global climate change would be to the same degree in both species.

After the F_1_ progeny of both species hatched from the pupal case, they were held under either treatment conditions for additional exposure as an adult (Egg Larva Pupa Adult) or switched to the control temperature for developmental exposure only (Egg Larva Pupa). This resulted in a total of 4 treatment groups - (i) reared at control and kept in control (C-C), (ii) reared at control and switched to high temperature (C-H), (iii) reared in high temperature and switched to control (H-C), and (iv) reared in high temperature and kept in high temperature (H-H).

### 2.3. Reproduction

Additional crosses were set up in enclosed insect breeding cages (ProLab, 2022), to measure the number of eggs, pupae, larvae, and adults. Each species and treatment combination was replicated six times, with ~ 5 females per replicate in Petri dishes containing molasses-agar media with a few granules of yeast on top. To ensure mating, virgin females and males were collected and allowed to mate for 48 h in polypropylene enclosures, beginning at age 1-day post-eclosion for *D. melanogaster* and 6 days post-eclosion for *D. pseudoobscura*. These ages were selected based on sexual maturation (see above). After 48 h, males were discarded, and females were transferred to the enclosure in insect breeding cages. Flies were transferred to new Petri dishes twice per day for 5 days, and the eggs in each Petri dish were counted immediately after the transfer. Then, the larvae, pupae, and adults in each Petri dish were counted daily. Each Petri dish was maintained at the adult parental treatment temperature for a total of 20 days to track reproductive output at each stage of development. Five different measurements were taken-(1) number of eggs laid, (2) number of hatched eggs, (3) number of larvae, (4) number of pupae, and (5) number of adults. The adults were removed after eclosion to avoid double counting. In the data analysis, it was determined that the count of larvae was not reliable likely due to them moving throughout the Petri dish causing an overcounting of this stage. Therefore, only data for eggs, pupae, and adults were reported.

### 2.4. Physiology

Thermal Tolerance. Critical thermal maximum (CT_max_) was used as a proxy for thermal tolerance. 10-day old flies were tested in a custom-designed microprocessor-controlled incubator that heats or cools at a constant rate (Hu & Appel, 2004). Flies were individually weighed and then confined in small chambers and an additional opened chamber with water was placed inside the incubator to prevent and control for desiccation. Temperatures were increased at 0.1°C per minute while observing knockdown. CT_max_ was defined as the highest temperature at which the fly was knocked down, unable to flip back up, yet able to recover after knockdown (following methods in (Sponsler and Appel, 1991). A minimum of 15–20 individuals per species per treatment per sex were used to determine thermal tolerance based on the CT_max_ measurements.

Respiratory Quotients (RQ). RQ defined as the volume of CO_2_ produced over the O_2_ consumed was measured on individual flies at 10 days old (post-eclosion), which is the time that, according to several studies, metabolic rates remain relatively constant in *Drosophila* (Arking et al., 1988; Hulbert et al., 2004; Mockett et al., 2001; Promislow & Haselkorn, 2002; Van Voorhies et al., 2003; Van Voorhies et al., 2004). Preliminary experiments were conducted on *D. pseudoobscura* to confirm that metabolic rates were relatively constant at 10 days in this species as well (see Supplemental Methods and Fig. S1). RQ was measured using closed-system respirometry as described by DeVries and Appel (2013). Briefly, flies were weighed pre- and post-incubation. Pre-weighed flies were placed in individual 1 ml syringes used as respirometry chambers. The chambers were flushed with dry, CO_2_-free air, sealed, and incubated in the dark for 4 h at the treatment temperature, but no more than 5 h to prevent additional stress due to starvation. Post-incubation, an air sample (0.25 ml) from each chamber was injected into a respirometry system and the data was recorded and analyzed using ExpeData software (Sable Systems, Henderson, NV, USA). The calculations were performed by converting the data into units of ml/minutes, then the peaks of both, CO_2_ and O_2_ were integrated and finally divided by body mass, to calculate the total CO_2_ production or O_2_ consumption per chamber. RQ was then calculated by dividing the CO_2_ produced by the O_2_ consumed. A minimum of 10 individuals per species per treatment per sex was used to determine metabolic rates.

### 2.5. Development

Stages of Oogenesis. Preliminary studies were performed to select two stages of oogenesis (see Figures S2–S3), early and late, for each species and treatment to compare the effects of each treatment on different time points. Ovaries from both species were dissected and stained with 0.5 % Toluidine blue as described in Abrams et al. (1993), and the stages of the oocytes were characterized at different time points until maturation age. From these slides, early and late time points (age in days post-eclosion) were selected. Dissections of the adult flies for *D. melanogaster* were performed starting at age 0 h and every 6 h for 30 h, while for *D. pseudoobscura*, dissections were made at age 0 days and daily for 7 days, due to the difference in time of sexual maturation between both species. The description of oocyte developmental stages by Jia et al. (2016) was used to define the time points, the early time point was defined as consisting of only oocytes in stages 1–10, and the late time point was defined as having all stages of oogenesis present. For *D. melanogaster* at 25°C, the selected time points were 1 and 4 days for control temperature, and for high temperature, 0.25 and 2 days. For *D. pseudoobscura*, for both treatments, the selected time points were 2 and 7 days.

Based on these selected time points, ovaries were collected from independent replicate groups of females per time point and per treatment. Only flies from 2 of the 4 treatment combinations were used, control (C-C) at 25°C and 20.5°C for *D. melanogaster* and *D. pseudoobscura* respectively, and high temperature (H-C) at 29.5°C and 25°C for *D. melanogaster* and *D. pseudoobscura* respectively. The tissue was then stained with the fluorescent stain DAPI (Vectashield with DAPI) and fluorescein-12-dUTP, using the DeadEnd^™^ Fluorometric TUNEL System from Promega and following the protocol described in Meehan et al. (2015). High-resolution pictures were recorded using the ZEISS Axiocam 503 microscope camera (Courtesy of Dr. Buckley and Dr. Range, Department of Biological Sciences, Auburn University), and analyzed using the biological picture analysis software Fiji (Schindelin et al., 2012) to identify the variability of the developmental stages of the ovarioles at different time points and compare the level of apoptosis present at specific developmental times for each treatment.

**Fig. 2.**
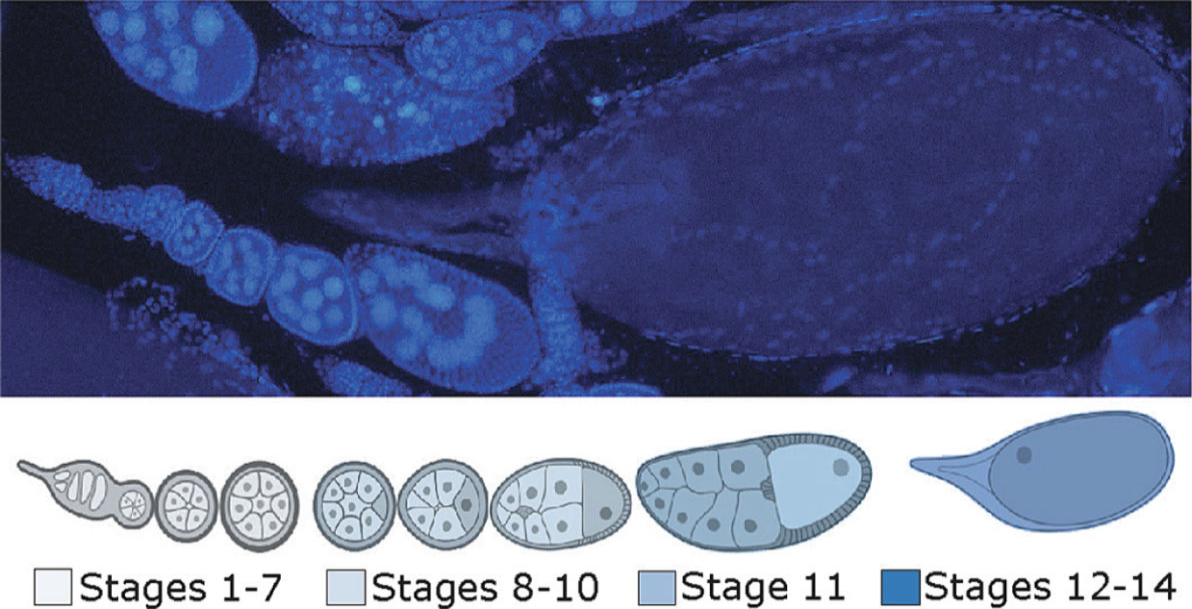
Comparison of the scheme of the development of each egg chamber and the pooled stages.

Image Analysis. Using the Oocyte developmental stages description by Jia et al. (2016), and the extension Fiji of the software ImageJ (Schindelin et al., 2012), the stage of development of all the visible egg chambers per individual was identified and corrected with size. The identified samples were then pooled into four developmental time points, stages 1 – 7, 8 – 10, 11, and 12 – 14 (Fig. 2).

### 2.5 Statistical Analysis.

Using R v.4.0166, each response variable was compared separately across treatments using the function ‘lmer’ from the “lme4” package (v.1.1–34, Bates et al. (2014)) to perform a linear mixed-effects model. For the physiological measurements, sex, treatment, species, and their interactions were included in the model as fixed effects while vial number was included as a random effect (e.g., *Bodymass* (1 |*vial*) + Treatment*Species*Sex). For fecundity, only females were tracked, and thus day, treatment, and the interaction were modeled as fixed effects. For models with a significant treatment effect, we then conducted a posthoc and contrast analysis, using the R package “emmeans” (v. 1.5.5–1, (Lenth and Lenth, 2018), to determine the significance across treatments for each response variable. The stages of oogenesis were compared across treatments with a negative-binomial regression mixed-model, using the “lme4” (v.1.1–34, Bates et al., 2014) and “car” (Fox et al., 2012) R packages. Stage, time point, treatment, and species were used as fixed effects with both replicate and ovariole as random effects (e.g., *Number Oocyte* (1 |*Ovary*) + (1 |*Ovariole* ) (1 |*Ovary* : *Ovariole*) + Stage*Timepoint*Treatment* *Species*). The presence or absence of TUNEL was analyzed using a binomial generalized linear mix model, and stage, time point, treatment, and species were considered as fixed effects. All the figures were plotted using the R packages “ggplot2” (Wickham, 2011), and “ggpubr” (v.0.6.6, Kassambara and Kassambara, 2020).

### 2.6. Data availability.

All the raw data, code, and additional model tables for this experiment are publicly available on GitHub. A static release of the repository is available via Zenodo (Rivera-Rincón & Stevison, 2024). All figures were created with Biorender.com.

## 3. Results

A combined total of 6,102 flies were collected from the 15 replicates from both species, consisting of 2,526 *D. melanogaster* and 3,576 *D. pseudoobscura* from all treatments. The physiological measurements for each treatment included CT_max_, body mass, CO_2_ production, and O_2_ consumption, as well as fecundity and oogenesis stages. Only females from 2 of the 4 combinations of treatments were evaluated for oogenesis stages, control (C-C), and high temperature (H-C).

### 3. 1 Fecundity.

**Fig. 3.**
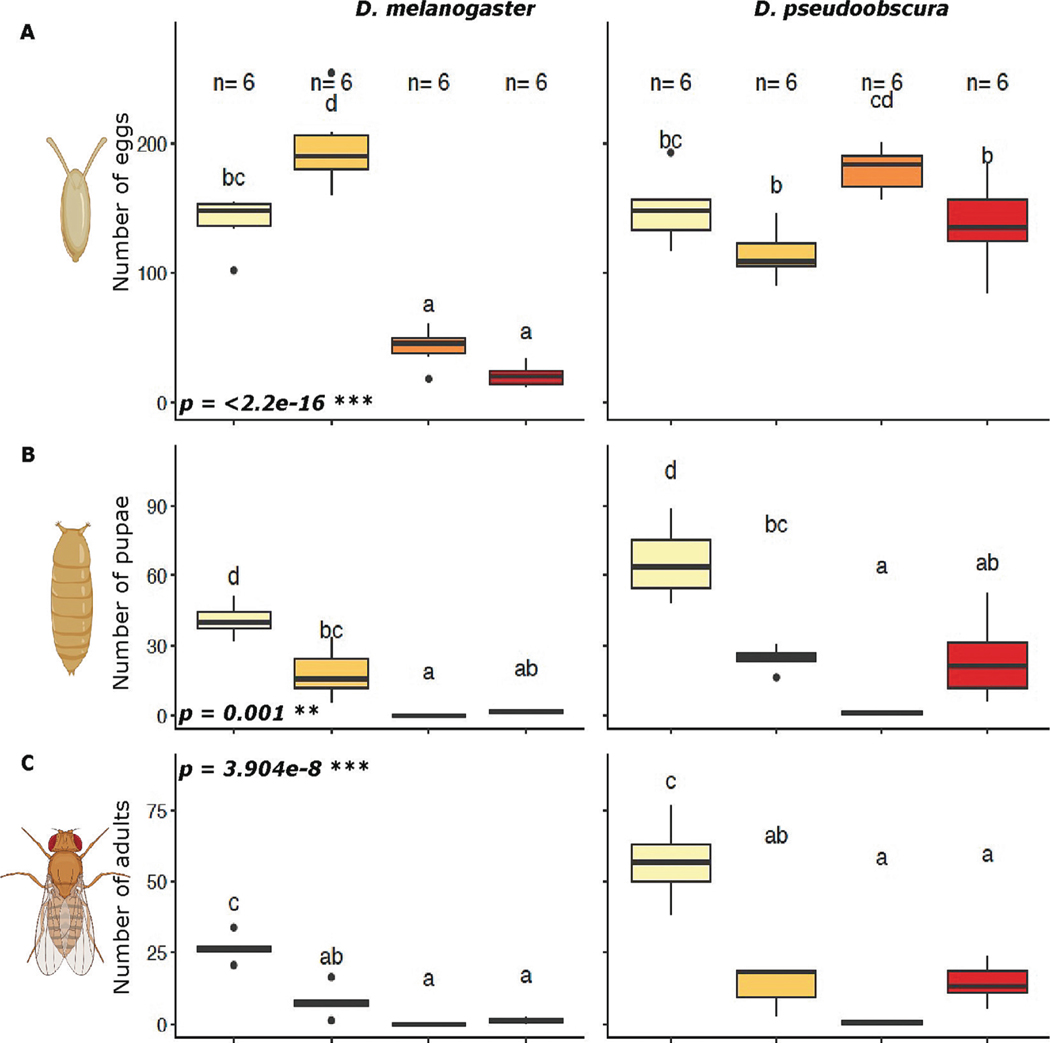
Box plots of three developmental stages with a side-by-side comparison between both species, *D. melanogaster* (~11 days development under control) on the left and *D. pseudoobscura* (~21 days development under control) on the right. The p-values in bold indicate significant differences due to treatments and the letters are the results of the posthoc test. A, the total number of eggs per female per day, over 5 days. B, the total number of pupae. C, the total number of adults.

A total of 209 females were tested, with 103 females for *D. melanogaster* and 106 females for *D. pseudoobscura*. The number of eggs laid per female showed a significant difference due to species (p = 9.22e-12), treatment (p=< 2.2e-16), and their interaction (p=< 2.2e-16). *D. melanogaster* showed significant differences in the survival of the different developmental stages due to treatment (p < 0.0001). Females under the C-H treatment presented the highest number of eggs laid per female, per day among treatments and species (~197 eggs). With 41.4 % and 84.8 % more eggs than the control and the other treatments respectively, whereas the number of pupae and adults was 97 % less than the control. Treatments H-C (42 eggs) and H-H (21 eggs) presented the lowest number of eggs per female among treatments and species and the numbers of pupae and adults dropped to 0 in both of these treatments (Fig. 3A–C, left). The number of pupae and adults under the H-C treatment in *D. pseudoobscura,* like *D. melanogaster,* drastically decreased to 0. However, *D. pseudoobscura*, contrary to *D. melanogaster,* had the highest number of eggs laid among treatments when reared under the H-C (~180 eggs) while the lowest number of eggs laid was under the C-H treatment (~114 eggs) (Fig. 3, right).

### 3.2 Physiology.

**Fig. 4.**
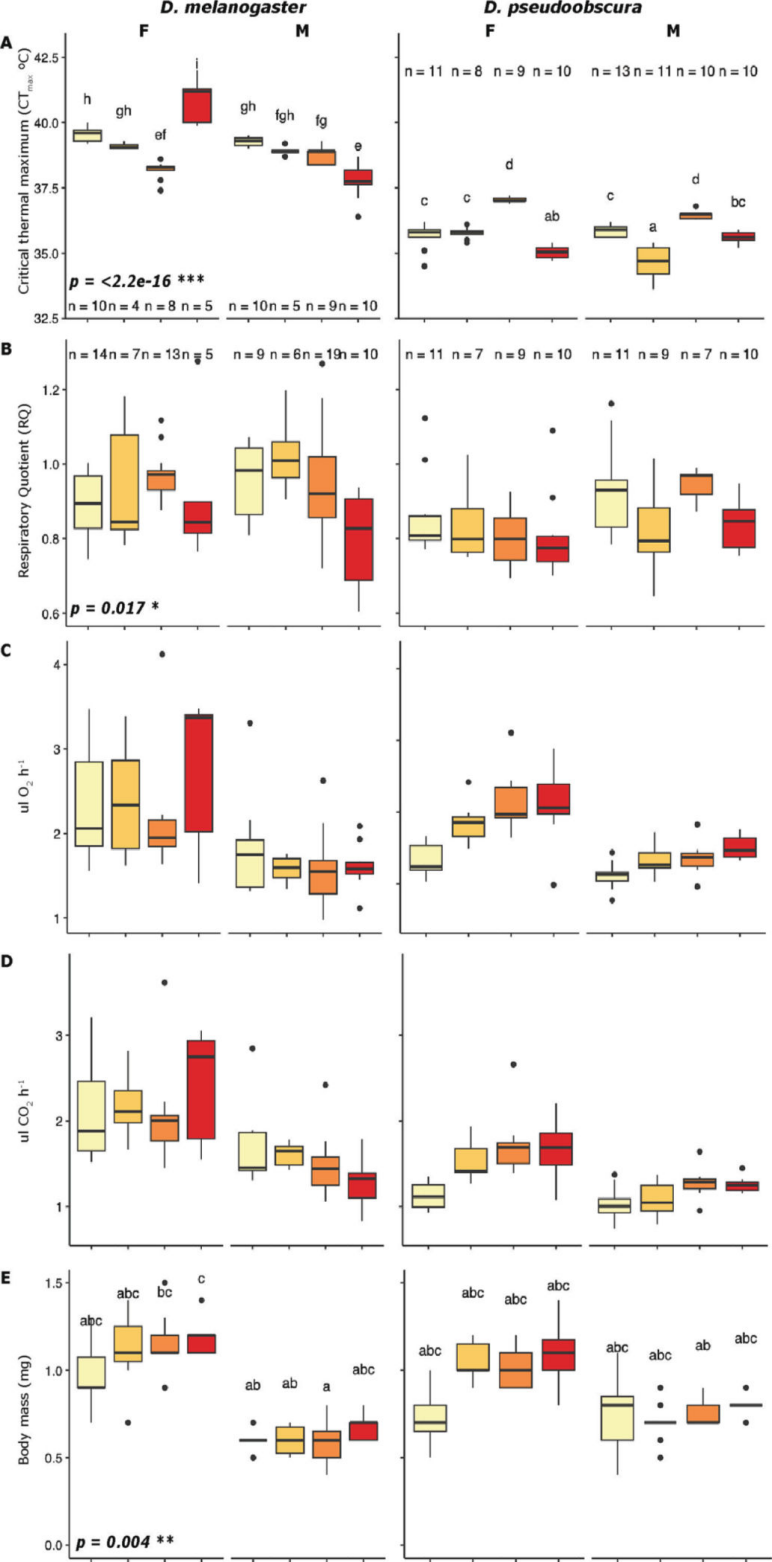
Box plots of physiology measurements with a side-by-side comparison between both species and sex, *D. melanogaster* on the left and *D. pseudoobscura* on the right. The p-values in bold indicate significant differences due to treatments and the letters are the results of the posthoc test. Number of individuals are for B, C, D, and E panels are the same, and are only described in panel B for reference. A, CT_max_. B, respiratory quotient (RQ). C, O2 consumption. D, CO2 production. E, body mass.

CT_max_ in both species was significantly different due to treatment (p < 2.2e-16), species (p = 1.88e-09), sex (p < 2.2e-16), and its interactions, including treatment:sex:species (p < 2.2e-16). Specifically, *D. melanogaster* females presented a CT_max_ of 40.88 °C under H-H, the highest among species, treatments, and sex, while males under the same treatment exhibited a CT_max_ of 37.76 °C. Under the H-H treatment, females and males had a difference of 3.1 °C in CT_max_ (Fig. 4A, left), as opposed to the other treatments, which differed by < 1 °C between sexes. Females were significantly different in CT_max_ for the treatments H-C and H-H when compared to the control (C-C). Similarly, females from the C-H treatment showed significant differences in their CT_max_ when compared to H-H and H-C treatments. Only males showed significant differences for H-H treatments when compared to any of the other treatments. *D. pseudoobscura* showed significant differences in CT_max_ for the H-C treatment compared to any other treatment for both sexes. Females under the H-C treatment had the highest CT_max_ (37.04 °C) within treatments and sex for the species (Fig. 4A, right).

Measures of Respiratory Quotient (RQ) followed the patterns observed in previous studies for *Drosophila* at control temperatures (Djawdan et al., 1996; Simmons & Bradley, 1997; Van Voorhies et al., 2004) with an RQ of around 0.95 for C-C for both species. Significant differences were observed in RQ due to species (p 0.0003) and treatment (p 0.01), but none was observed due to sex or any of the interactions. However, *D. melanogaster* males, under the H-H and C-H treatments, exhibited an RQ of 0.79 and 1.02 respectively, the lowest and highest values between sexes, species, and treatments (Fig. 4B). Similarly, consumption of O_2_ did not significantly vary due to species, treatment, or sex, but contrary to RQ the highest O_2_ consumption (2.73 ulO_2_h—1) was observed in females from *D. melanogaster* under the H-H treatment while the lowest O_2_ consumption (1.1 ulO_2_h—1) was observed in *D. pseudoobscura* males under C-C conditions, with a difference of 1.63 ulO_2_h—1 (Fig. 4C). Production of CO_2_, was not significantly different due to species, treatment, or sex, but showed similar patterns as O_2_ consumption, in both species due to treatment. However, while *D. melanogaster* reduced its amount of CO_2_ produced in H-C and H-H by 0.6 ulCO_2_h—1 compared to C-C, *D. pseudoobscura* increased the amount of CO_2_ produced in H-C by 0.45 ulCO_2_h—1 and H-H by 0.38 ulCO_2_h—1, when compared to C-C (Fig. 4D).

When comparing body mass, significant differences due to treatment (p = 0.004), sex (p= < 2.2e-16), and the interaction species:sex (p=<0.001) were observed. *D. melanogaster* had the highest and lowest values of body mass among the two species. Females under H-H conditions exhibited an average body mass of 1.2 mg, while males under H-C, exhibited an average of 0.58 mg, a difference of 0.62 mg (Fig. 4E, left). A post hoc analysis showed significant differences specifically between females and males reared under H-C treatment (p = 0.02). Similarly, *D. pseudoobscura* presented the highest body mass in females under H-H treatment (1.1 mg), while males under H-C, exhibited the lowest body mass among all the treatments and sexes, for the species (0.7 mg), with a difference of 0.4 mg between both of them (Fig. 4E).

### 3.3 Development.

**Fig. 5.**
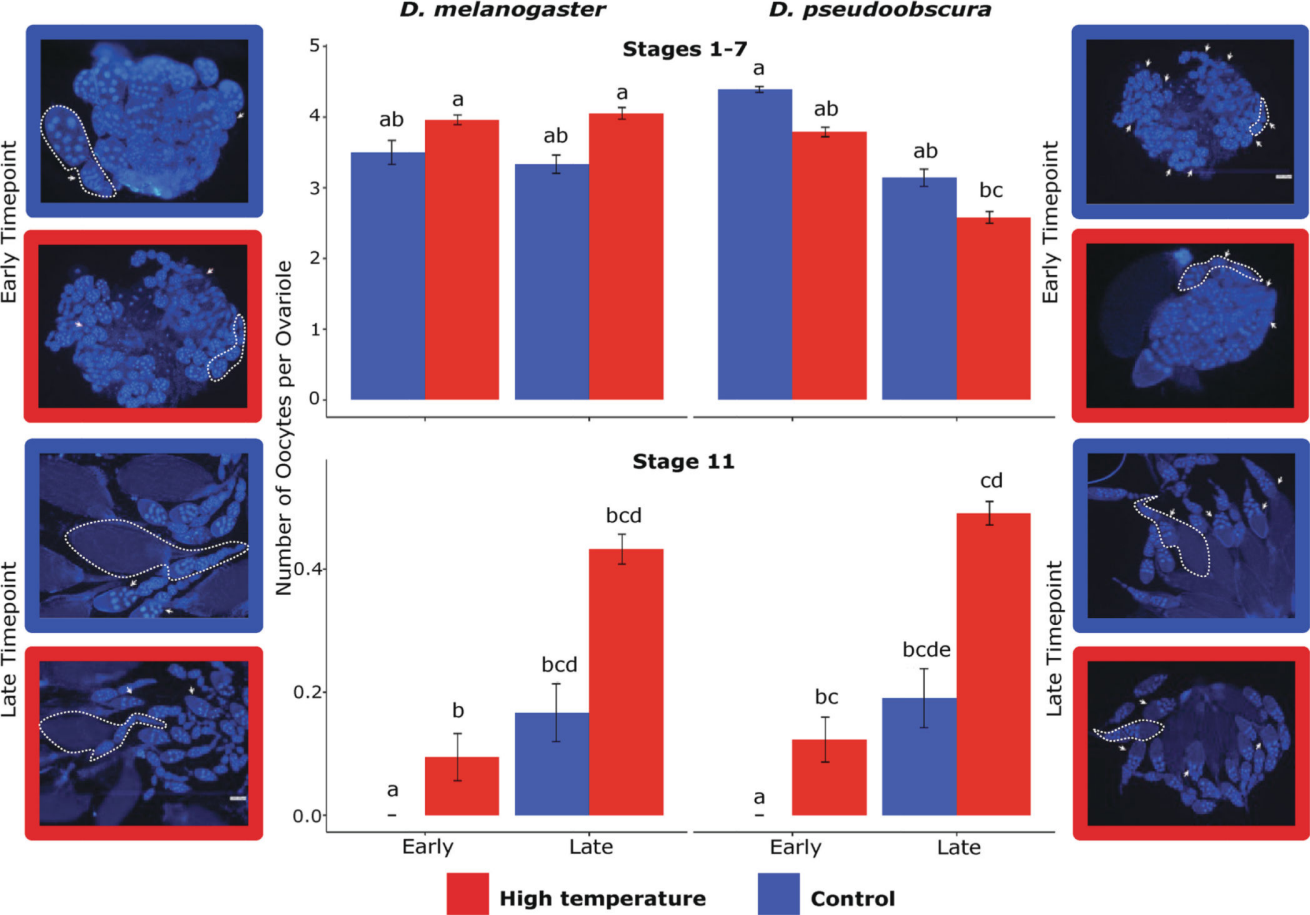
Number of oocytes per ovariole, at early and late time points for stages 1–7 (top) and stage 11 (bottom), with a side-by-side comparison between both species, *D. melanogaster* on the left and *D. pseudoobscura* on the right, and letters are the results of the posthoc test. On each side a representative picture of the ovary stained with DAPI. For stages 1–7 only early time points are represented, and for stage 11, only late time points are represented. White arrows pointing at selected oocytes of either stages 1–7 (top) or stage 11 (bottom) and dotted lines showing individual ovarioles with varying stages of oocytes for each species.

**Fig. 6.**
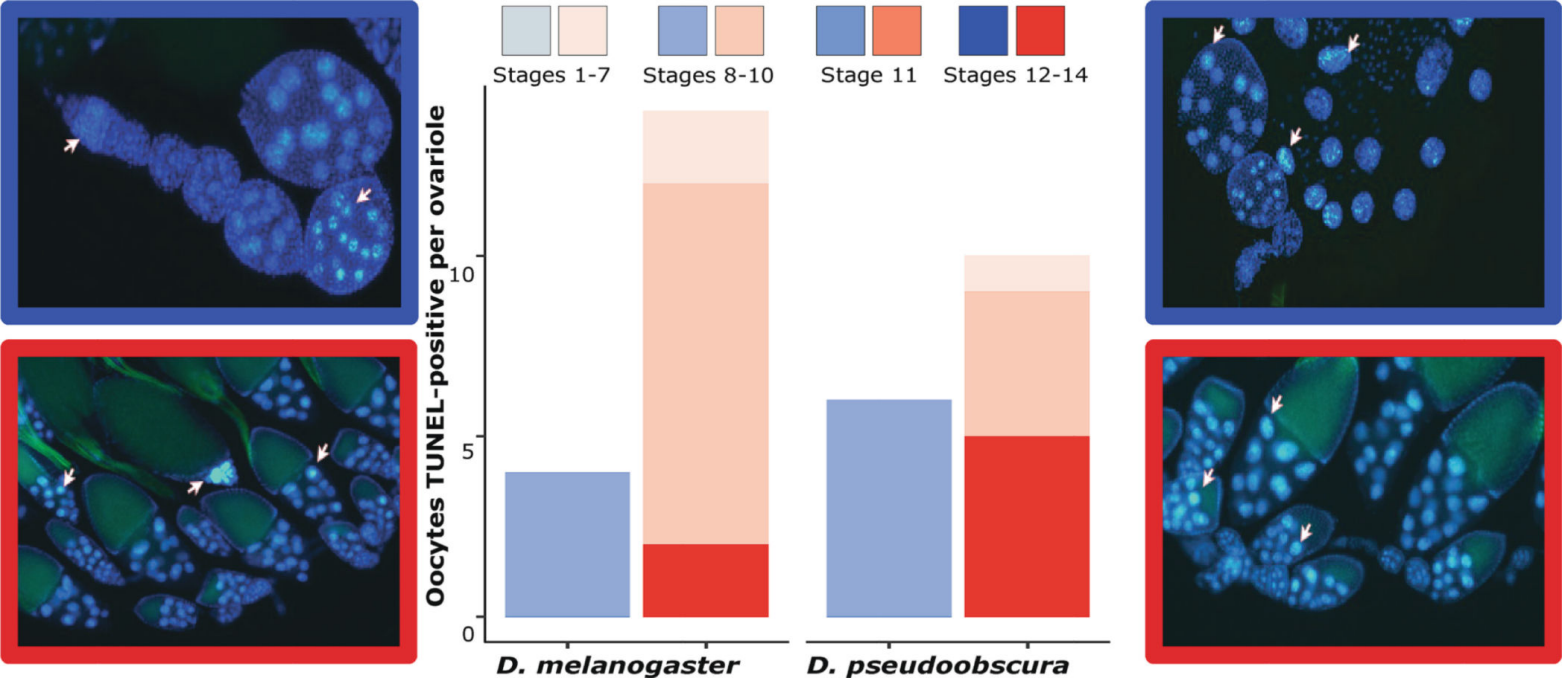
Number of TUNEL-positive oocytes per ovariole, at early time point, with a side-by-side comparison between both species, *D. melanogaster* on the left and *D. pseudoobscura* on the right. Color gradients for control and high temperature between the four different developmental stages. On each side a representative picture of the ovary stained with DAPI (royal blue) and TUNEL (bright green), with white arrows pointing at selected TUNEL-positive oocytes for control (C-C, blue), and high temperature (H-C, red).

Both species exhibited accelerated oocyte development under high temperature, resulting in significant differences between stages (p=< 2e-16), and some of the interactions between species, stages, treatments, and time points, including stage:timepoint: species (p = <0.001), stage:timepoint:treatment (p = < 2.2e-16), and stage:timepoint:treatment:species (p = 8.7e-14). Specifically, we observed an increase in the number of oocytes present at the early time points in stages 8–10, 11 (Fig. 5, bottom), and 12–14 due to treatment (H-C), when compared to control (C-C), where stages 11, and 12–14 were expected only for late time points. Contrarily, for the late time points within the same stages, both treatment groups presented comparable numbers of oocytes, except for stage 11 (Fig. 5, bottom) in *D. pseudoobscura*, where the high-temperature treatment resulted in a higher number of oocytes at this stage compared to the control (C-C). In *D. melanogaster,* oocytes were only observed in stages 1–7 (Fig. 5, top), 8–10, and 11 (Fig. 5, bottom), at the late time point in the control treatment (C-C), while at the same time point under high temperatures (H-C), all stages were visible. Notably, stages 1–7 in both early and late time points showed an increase of 1 to 2 more oocytes per ovariole in *D. melanogaster* for flies reared under H-C (Fig. 5, top left). On the contrary, *D. pseudoobscura* exhibited the opposite trend, with a decrease of 1 to 2 oocytes per ovariole under the high-temperature treatment (Fig. 5, top right). The number of TUNEL-positive oocytes was significantly different due to species (p = 1.11e-10), treatment (p = < 2.2e-16), time point (p = 3.66e-06), stage (p = 3.98e-07), and some of the interactions including treatment:species (p = 4.66e-05) timepoint: treatment:species (p = < 2.2e-16), and stage:timepoint:treatment:species (p = < 2.2e-16). Due to the acceleration in oogenesis, stages 11, and 12–14, were visible in the early time points, in which the number of TUNEL-positive oocytes from stages 8–10, 11, and 12–14, increased in high-temperature stress (H-C) for both species (Fig. 6). For *D. melanogaster* this increase was up to 2 oocytes per ovariole for stages 8–10 and 11, and up to 10 oocytes per ovariole in stages 12–14 (Fig. 6, left), whereas for *D. pseudoobscura*, stages 8–10 increased in 5 oocytes per ovariole, stage 11 in 0.8 oocytes per ovariole and stages 12–14 in 4.2 oocytes per ovarioles (Fig. 6, right). The late time points showed an increase in the number of TUNEL-positive oocytes in all stages at high temperature (H-C), only in *D. pseudoobscura.*

## 4. Discussion

Temperature spikes are expected to increase in severity, regularity, and duration, with some studies indicating changes at a faster rate than previously predicted (IPPC, 2007). These temperature changes increase the likelihood of species with short lifespans to experience higher temperatures during one or more of its developmental stages (Zhang et al., 2015). Despite both strains having a comparable longitudinal origin, we predicted that the *D. melanogaster* strain Canton-S, would be less susceptible to thermal stress than the *D. pseudoobscura* strain MV2–25, due to the cosmopolitan distribution of the species as a whole. However, we found the opposite, with *D. melanogaster* exhibiting a lower overall fecundity (egg, pupae, and adult outcome), higher decreases in CT_max_, and higher oocyte apoptosis when exposed to high temperatures during development, compared to *D. pseudoobscura*.

CT_max_ values in *D. melanogaster* showed a decrease as big as 0.98 °C under high temperature treatments, while *D. pseudoobscura*, showed a decrease of 0.54 °C. Body mass measurements showed a lower increase (0.152 mg) for *D. melanogaster*, while *D. pseudoobscura* presented almost double body mass increase (0.214 mg). Body mass has been described to be impacted under thermal stress, as a result of depletion of body fat content and increased energy demands (Klepsatel et al., 2016). The lower changes in body mass and the higher O_2_ consumption in *D. melanogaster* are consistent with a higher energy demand under thermal stress, compared to *D. pseudoobscura.* These differences may be explained by the shorter lifespan of *D. melanogaster* compared to *D. pseudoobscura,* and are discussed in more detail in section 4.2 below.

Previous research has shown that species can respond differently to increasing temperatures at both broad and local scales, depending on other factors such as degree of exposure and life history. However, it is unclear if a relationship between life history and the degree of stress response is present between these species. Similar to previous studies evaluating the effects of thermal stress on fecundity during either development or adulthood (Krebs and Loeschcke, 1994; Melicher et al., 2021; Sisodia & Singh, 2006; Stazione et al., 2021), our experiment showed an overall decrease in fecundity for both species. Notably, *D. melanogaster* exhibited the lowest fecundity rates across all stages and temperature treatments. We observed distinct patterns of thermal tolerance changes for each treatment and species, as well as the accelerated progression through oogenesis and increased apoptosis of oocytes in specific developmental stages.

### 4.1. Reproductive output of earlier developmental stages is more sensitive to parental high temperature treatment

Both species experienced a reduction in their overall reproductive capacity but in distinct ways. It is important to note that our reproductive measurements refer to fecundity following treatment as opposed to survival during treatment (see methods). *D. melanogaster* exhibited greater susceptibility to the impacts of high temperatures when exposed during all the stages of development (egg + larvae + pupae), regardless of the additional exposure as an adult (H-C and H-H), exhibiting lower fecundity across all the stages of development. On the other hand, *D. pseudoobscura* was most significantly affected when exposed to high temperatures during all stages of development (egg + larvae + pupae), only if the adult was not exposed (H-C). Heat stress during early developmental stages is not shown to impact fecundity, when exposed to high temperatures for a short period, while long-term exposure to moderate temperatures has been shown to impact fecundity and longevity, only when the adult was not exposed (Zhang et al., 2015). The differences in the effects of high temperatures on fecundity, seems to depend on whether repair mechanisms have the opportunity or time to act, regardless of the stage(s) exposed. The short developmental time of *D. melanogaster* could reduce the ability of repair mechanisms to act before or during the adult stage.

Previous studies have demonstrated a decrease in the number of eggs laid by *D. melanogaster* after exposure to high temperatures. Still, oogenesis is a process regulated by several factors (i.e., hormonal, genetic, metabolic, etc.), and impairment in any of these can affect the quantity and quality of eggs produced (Greenblatt et al., 2019; Greenblatt & Spradling, 2018). Interestingly, the C-H treatment in *D. melanogaster* and the H-C treatment in *D. pseudoobscura* both showed an increase in egg production compared to the control (Fig. 3A), perhaps as a way to respond to temperatures nearer to the biological temperature threshold (Evans et al., 2018).

Reparative mechanisms in oogenesis have been suggested in *D. melanogaster*, where temperature changes seem to trigger the activation of the DNA-damage-checkpoint and modulate *P* element activity in germline stem cells allowing DNA-damage repair and subsequent progression past associated checkpoints within a 4 day period (Moon et al., 2018). These mechanisms indicate the presence of one or several constraints that limit the capacity to respond to changes in temperature. Although the nature and limitations of such constraints are not yet fully understood, the differences observed in our experiment in egg-to-adult reproductive output in these two species with ~ 10 days difference in developmental times could suggest a constraint related to time. There are trade-offs between faster developmental times and other life-history traits, and time could potentially influence the effectiveness of the activation and regulation of the DNA-damage checkpoint (Sørensen & Loeschcke, 2004). Moreover, despite the broader geographic distribution of *D. melanogaster*, *D. pseudoobscura* was more tolerant of high temperatures during both early developmental life stages and in adults (H-H), with the number of eggs, pupae, and adults following closely the patterns of C-H. These results suggest that *D. pseudoobscura* may be undergoing a process of acclimation during earlier developmental stages. Alpine species can experience dramatic temperature fluctuations on a daily and seasonal basis than cosmopolitan species (Kinzner et al., 2018). If this is the case for alpine species, then it may explain the greater tolerance to high temperatures observed here for *D. pseudoobscura*, compared to the cosmopolitan species *D. melanogaster*.

### 4.2. Sex differences in physiological response to thermal stress vary between species

In contrast to our main hypothesis, we found that *D. melanogaster* showed a greater overall response to thermal stress than *D. pseudoobscura*. Specifically, for CT_max_, we observed a difference between means of ~ 1 °C between sex and treatments in *D. pseudoobscura*, compared to the almost 4 °C observed *in D. melanogaster*. This result could be attributed to the differences in development time between the species, where *D. melanogaster* develops faster than *D. pseudoobscura*. The difference in development time is more pronounced under thermal stress, resulting in reduced time for *D. melanogaster* to initiate mechanisms that might lead to higher tolerance of extreme temperature shifts. Additionally, an inverse pattern was observed between the sexes in both species: *D. melanogaster* females showed bigger changes in CT_max,_ whereas in *D. pseudoobscura,* it was the males that exhibited greater changes. Previous studies showed similar results, where *D. melanogaster* males exhibited greater heat stress adaptation compared to females (Folk et al., 2006; Khazaeli et al., 1997). The differences in response between sexes could be explained by sex-specific patterns previously described in *Drosophila* for required genes in stress response (Moskalev et al., 2011; Tower et al., 2020). In *Drosophila*, sex determination pathways seem to regulate sex-specific patterns in stress adaptation, where females have been described to preferentially require more genes for stress response than males (Moskalev et al., 2011; Moskalev et al., 2012).

The assessment of critical thermal limits has been widely conducted in various species to understand their responses to climate change. In *Drosophila*, the flexibility of thermal tolerance has been extensively studied, considering factors like acclimation, developmental temperature, resource availability, and life history traits, including seasonal variations. Studies of *D. melanogaster* at control temperatures have reported similar findings to our results, with thermal maxima around 40 °C reported for multiple stocks (Jørgensen et al., 2020; Lecheta et al., 2020; Rolandi et al., 2018). However, it is important to note, that some studies have shown varying results. Kellermann et al. (2017) observed values of CT_max_ > 40 °C for flies either aged 5 or 25 days under control conditions and different temperature treatments. Considering that many of these studies pool individuals of different sexes, perhaps differences in thermal maxima across studies for the same species could be due to the influence of other factors, such as age and sex that have been shown to have an impact on CT_max_ (Kristensen et al., 2019). Similarly, the magnitude of the stressor can also have an impact on thermal tolerances, as demonstrated by Schou et al. (2017), where flies reared at either excessively low or high temperatures exhibited a lower CT_max_ than predicted by linear models. In the same way, experimental protocols vary significantly in the specific definition of CT_max_, leading to differences in the magnitude of physiological stress and its interpretation. Our study aimed to control for various factors that can influence thermal tolerances, such as sex, age, and rate of heating, in order to compare responses between sexes and two *Drosophila* species.

Although the observed patterns shown here in thermal tolerance between the species are opposite of each other, there is a trend, where treatments resulting in higher CT_max_ also exhibit a lower fecundity in terms of egg-to-adult viability in both species, which suggests a possible trade-off between constraints in fecundity and survival. Specifically, *D. melanogaster* presented the highest CT_max_ in females exposed to H-H (see Fig. 4A, left), and their fecundity was the lowest for the same treatment for the species (see Fig. 3, left). For *D. pseudoobscura*, the highest CT_max_ was for females under H-C treatment (see Fig. 4A, right), which in terms of fecundity was the only treatment that rapidly decreased to 0 as early as the pupae stage (see Fig. 3B, right). Previous studies have demonstrated that constraints on fecundity and survival vary along climate clines. Cosmopolitan species (i.e., leading range) tend to have greater constraints on fecundity, while more narrowly distributed species (i.e., trailing range) face higher constraints on survival (Rehm et al., 2015). These studies indicate that fitness is a product of systematic shifts within and between fecundity and survival traits, in response to environmental conditions (Kellermann & van Heerwaarden, 2019; Overgaard et al., 2014; Sinclair et al., 2016). Species often survive at suboptimal thermal ranges that are lower than those optimal for reproduction, causing temporal and/or permanent changes in the gain, use, and storage of different forms of energy (Scranton & Amarasekare, 2017). Additionally, both species showed significant differences in RQ due to treatment, while sex and species did not present significant differences, with RQ, O_2_ consumption, and CO_2_ production following the patterns in previous studies for *Drosophila* (Djawdan et al., 1996; Simmons & Bradley, 1997; Van Voorhies et al., 2004) under control conditions. These differences in responses between treatments suggest a potential shift in substrate oxidation that could influence the balance between fecundity and survival constraints in both species.

### 4.3. Acceleration in oogenesis due to high temperatures

Temperature accelerated oocyte development in both species and increased rates of apoptosis, particularly in the early stages of oogenesis, as observed at 0.25 and 2 days old post-eclosion for *D. melanogaster* and *D. pseudoobscura* respectively. While both species presented on average 19 more oocytes in stages 11 and 12–14, when exposed to high temperatures, TUNEL-positive oocytes were increased in both late and early time points in *D. pseudoobscura*, compared to *D. melanogaster* that showed an increase only in the late time points. As previously mentioned, oogenesis is a complex process that is controlled by many factors. Any modifications to these factors, such as changes in temperature, can lead to significant changes in the quantity and quality of oocytes. These changes may be directly related to the lower egg-to-adult viability observed across all treatments for both species and may cause dysfunction in regulatory processes or critical metabolic pathways (Gandara & Drummond-Barbosa, 2022; Sokolova et al., 2013).

This study offers valuable insights into the intricate impacts of increasing environmental temperatures and underscores the significance of assessing diverse components associated with fitness to characterize these impacts. By evaluating more than one response to stress, more effective predictions and conservation strategies can be made for species in the face of climate change. However, it is important to acknowledge that while this study involved two distinct species with comparable latitudinal origin and different geographic distributions, we used only a single inbred stock that may not have fully captured the extent of variation across different geographical populations of each species (Gaston & Chown, 1999; Schiffer et al., 2013). Therefore, to fully understand the associations between the diverse fitness components and the life history of each species additional work on more strains of each species will be required. Another caveat of our study is that we used inbred strains. Previous work has shown that often, field flies showed a lower heat resistance, compared to inbred stocks from the same location (Schiffer et al., 2013). Those differences in heat resistance have been attributed to carry-over effects associated with environmental effects, like the development of field flies under poor conditions. However, inbred stocks are reared under controlled laboratory conditions, minimizing different sources of variation that could affect the response of a species (Schiffer et al., 2013). Additionally, controlled conditions allow a more robust characterization of the effects in the response to specific conditions, like thermal stress which was a benefit of our approach. Still, to better understand the effects of climate change, future studies should consider the constraints within and between different fitness components and a more comprehensive representation of the natural populations of the model or indicator species.

During the preparation of this work, the first author used Grammarly and ChatGPT in order to improve the readability and language of the work. After using this tool/service, the first author reviewed and edited the content as needed and takes full responsibility for the content of the publication.

## Supplementary Material

MMC1

## Data Availability

All data is publicly available on GitHub and linked in the main manuscript file.
