# Supplementary material for: “A comparison of thermal stress response between Drosophila melanogaster and Drosophila pseudoobscura reveals differences between species and sexes”
Source: J Insect Physiol. Author manuscript; Available in PMC 2024 Apr 26. (PMC11048572; doi:10.1016/j.jinsphys.2024.104616)

Supplementary Material and Methods

Age selection for RQ in *Drosophila pseudoobscura*

Stocks. Fruit flies *Drosophila pseudoobscura* (wild-type, MV2-25) were growth and maintained at 21°C with a 12-hour light-dark cycle in an incubator and reared on standard cornmeal–sugar–yeast–agar media in polypropylene enclosures. For experiments, adult flies were placed into a fresh vial (sex-mixed) and discarded after 24 hours to prevent additional stress (Priest, 2007).

Metabolic rate measurements. RQ defined as the volume of CO_2 produced over the O_2 consumed was measured on individual flies at 10 days old (post-eclosion), which is the time that, according to several studies, metabolic rates remain relatively constant in *Drosophila* (Arking et al., 1988; Hulbert et al., 2004; Mockett et al., 2001; Promislow and Haselkorn, 2002; Van Voorhies et al., 2004, 2003). Preliminary experiments were conducted on *D. pseudoobscura* to confirm that metabolic rates were relatively constant at 10 days in this species as well (see Figure S1). RQ was measured using closed-system respirometry as described by DeVries and Appel (2013). Briefly, flies were weighed pre- and post-incubation. Pre-weighed flies were placed in individual 1 ml syringes used as respirometry chambers. The chambers were flushed with dry, CO_2-free air, sealed, and incubated in the dark for 4 hours at the treatment temperature, but no more than 5 hours to prevent additional stress due to starvation. Post-incubation, an air sample (0.25 ml) from each chamber was injected into a respirometry system and the data was recorded and analyzed using ExpeData software (Sable Systems, Henderson, NV, USA). The calculations were performed by converting the data into units of ml/minutes, then the peaks of both, CO_2 and O_2 were integrated and finally divided by body mass, to calculate the total CO_2 production or O_2 consumption per chamber. RQ was then calculated by dividing the CO_2 produced by the O_2 consumed. A minimum of 10 individuals per species per treatment per sex was used to determine metabolic rates. After eclosion, virgin females and males were collected and placed separately into fresh food and transferred every 7 days to fresh vials until assayed. To compare the changes in age, flies were assayed at 2, 5, 7, 10, and 20 days old.

All flies were incubated at 21°C for 4 hours to guarantee a good sample and to prevent stress due to starvation and its been related to cause major biochemical disturbances in lipids and other metabolic pathways (Chatterjee et al., 2014; Choi et al., 2015).

Supplementary figures

S1: Respiratory Quotient for D. pseudoobscura for 20 days

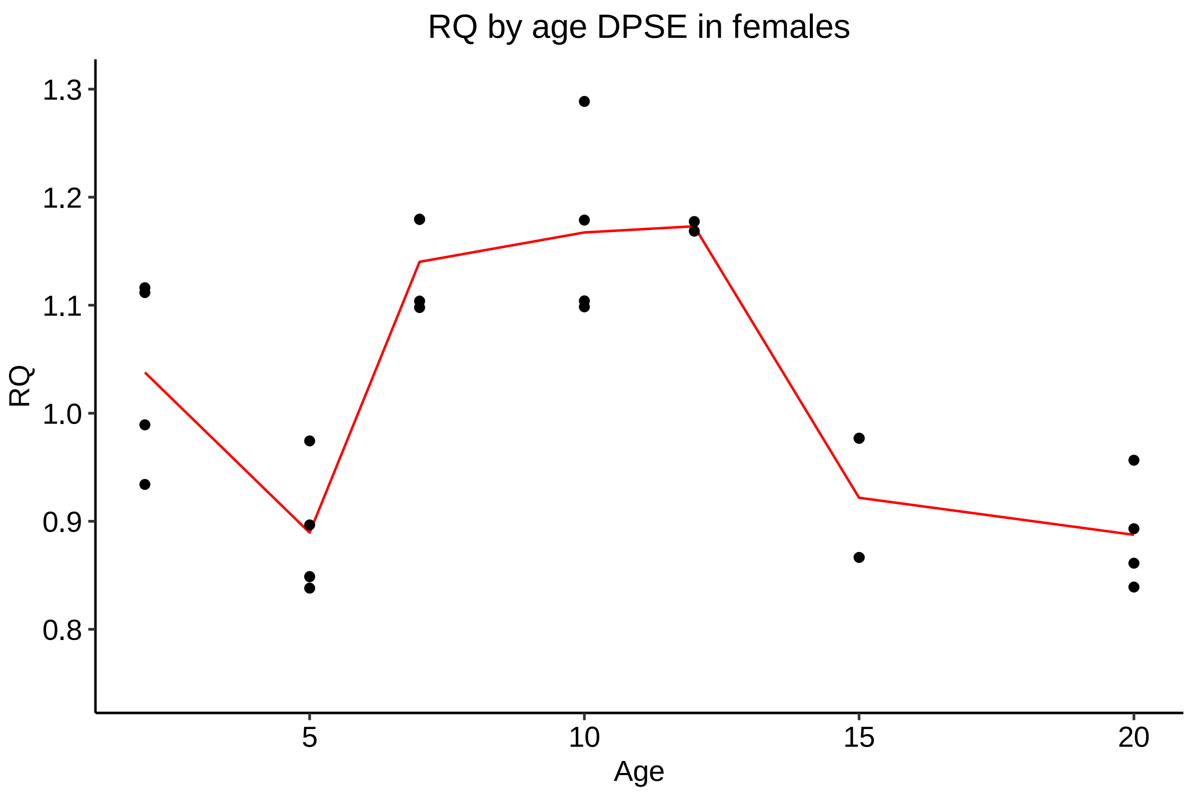

Supplementary Material

S2: Timeline of oogenesis under high temperatures and control for *D. melanogaster*.

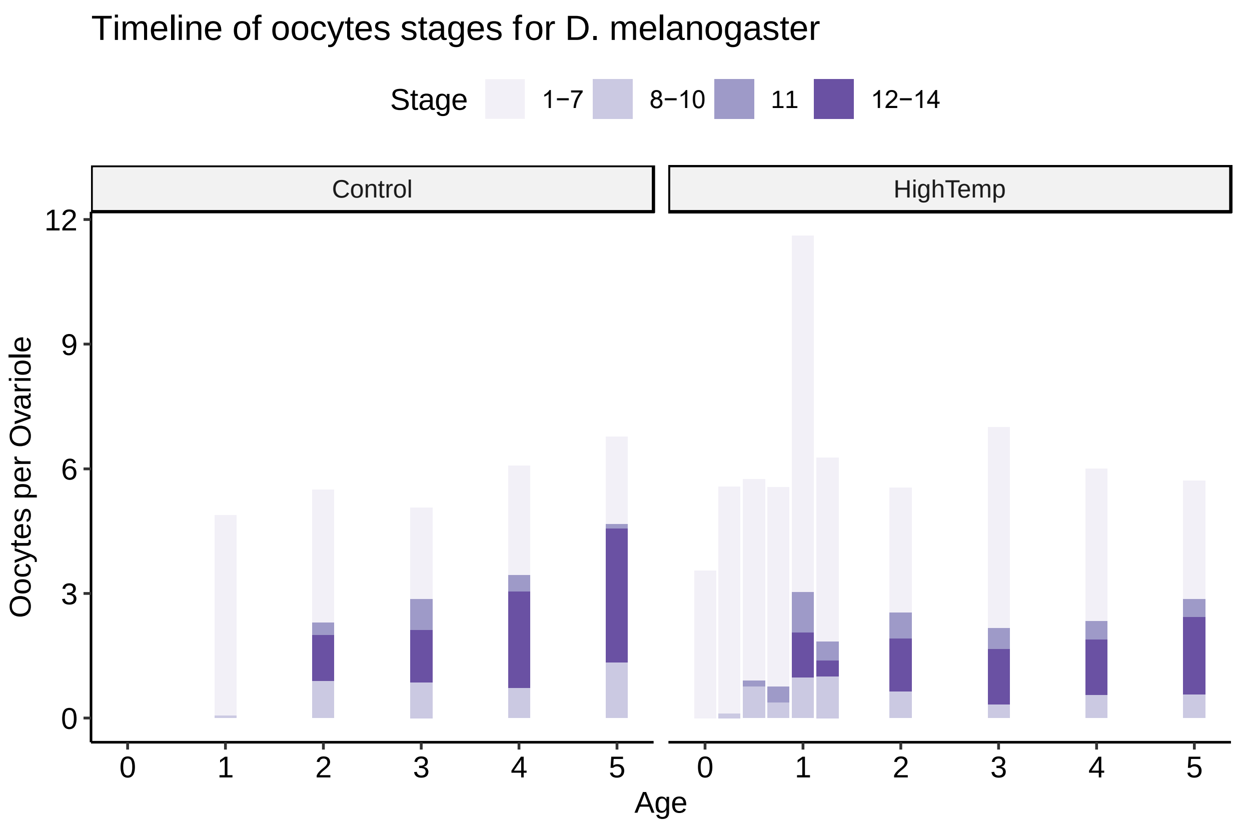

Supplementary Material

S3: Timeline of oogenesis under high temperatures and control for *D. pseudoobscura*.

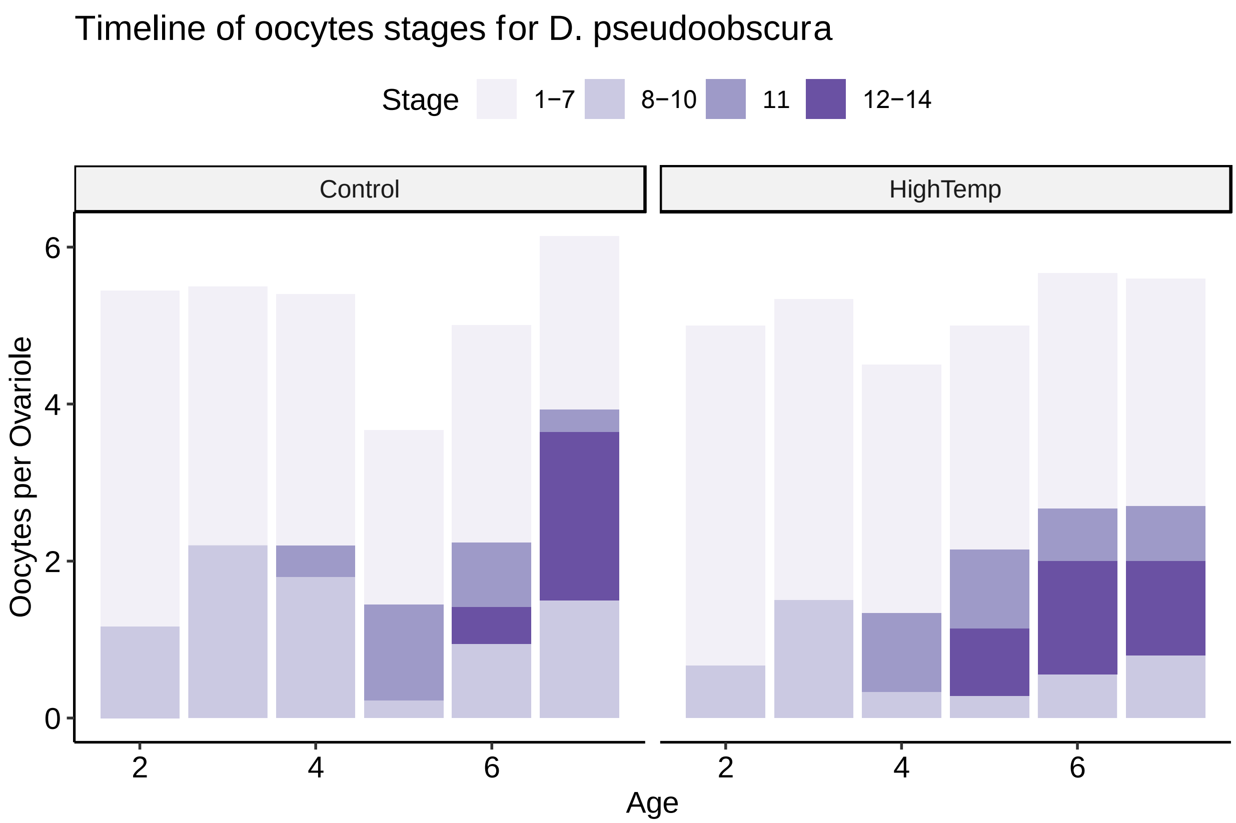

Supplement: MMC1 [file NIHMS1964827-supplement-MMC1.pdf]
